# Opacity and emergence: rethinking the role of phenomenological psychopathology through the prism of adolescence

**DOI:** 10.1192/j.eurpsy.2026.10332

**Published:** 2026-07-31

**Authors:** L. Madeira

**Affiliations:** Centro de Bioética e Humanidades Médicas, Faculdade de Medicina da Universidade de Lisboa, Lisboa, Portugal

## Abstract

**Abstract:**

Adolescence is a developmental epoch marked by vulnerability, emergence, and existential reorientation—a time when the self is being formed as much as it is being questioned. In this contribution, I argue for a critical reappraisal of the phenomenological approach within child and adolescent psychiatry, drawing from classical sources and contemporary insights. The aim is twofold: first, to question the risks of epistemic reductionism and diagnostic imposition in adolescent psychiatry; second, to explore how phenomenology might foster non-interpretative, heterological forms of clinical engagement with the adolescent subject. I begin by revisiting Karl Jaspers’ existential philosophy and methodological framework, including his emphasis on Verstehen (understanding) and Grenzsituationen (limit situations), to show how they remain crucial for situating adolescent suffering within a broader human context, rather than confining it within symptom-based taxonomies. I then turn to the concept of heterological empathy, which foregrounds the radical alterity of the other’s experience, especially pertinent in the adolescent who may not yet possess narrative clarity or diagnostic legibility. This requires clinicians to approach their young interlocutors not through projection or interpretation, but through a receptive stance that welcomes the foreignness of their world-making. Building on this, I propose a critique of epistemic injustice in adolescent psychiatry—how young people’s self-descriptions are often discounted or filtered through adult-centric and biomedical frameworks. This contributes to both diagnostic overreach and neglect, particularly in cases where ambiguity and developmental flux are mistaken for pathology. Phenomenological psychopathology is especially well-suited to rethinking adolescence not as a problem to be managed but as a threshold structure—a phase of becoming that is both unstable and meaningful. I highlight the potential for phenomenologically-informed interventions in this age group. These include dialogical spaces that support self-articulation, embodied therapeutic practices, and intersubjective frameworks that reorient treatment from symptom eradication to existential accompaniment. In conclusion, this keynote aims to demonstrate that the application of phenomenology in adolescent psychiatry is not merely a theoretical enrichment—it is an ethical imperative. By resisting the pressures of over-diagnosis and interpretive closure, phenomenology reasserts the adolescent’s right to opacity, ambiguity, and emergence. It reconfigures the clinical relationship not as a site of knowledge extraction, but as a shared space of meaning-making where both clinician and adolescent stand as co-inhabitants of a fragile, unfolding world.

**Disclosure of Interest:**

None Declared

